# Supplementary material for: Kalanchoe tomentosa: Phytochemical Profiling, and Evaluation of Its Biological Activities In Vitro, In Vivo, and In Silico
Source: Pharmaceuticals (Basel). 2024 Aug 9;17(8):1051. doi: 10.3390/ph17081051 (PMC11357309; doi:10.3390/ph17081051)
Supplement: Supplementary file 1 [file pharmaceuticals-17-01051-s001.zip › pharmaceuticals-3140682-supplementary.pdf]

Supplementary materials

Kalanchoe tomentosa: Phytochemical Profiling, and Evaluation of Its Biological Activities *In Vitro*, *In Vivo*, and *In Silico*

Jorge L. Mejía-Méndez [1,2,†], Gildardo Sánchez-Ante [3,†], Yulianna Minutti-Calva [2], Karen Schürenkämper-Carrillo [2], Diego E. Navarro-López [3], Ricardo E. Buendía-Corona [2], Ma. del Carmen Ángeles González-Chávez [1], Angélica Lizeth Sánchez-López [3], J. Daniel Lozada-Ramírez [2,*], Eugenio Sánchez-Arreola [2,*] and Edgar R. López-Mena [3,*]

[1] Programa de Edafología, Colegio de Postgraduados, Campus Montecillo, Carr. México Texcoco km 36.4, Montecillo 56230, Mexico; jorge.mejiamz@udlap.mx (J.L.M.-M.); carmeng@colpos.mx (M.d.C.Á.G.-C.).

[2] Departamento de Ciencias Químico-Biológicas, Universidad de las Américas Puebla, Ex Hacienda Sta. Catarina Mártir S/N, San Andrés Cholula 72810, Mexico; yulianna.minuttica@udlap.mx (Y.M.-C.); karen.schurenkamperco@udlap.mx (K.S.-C.); ricardo.buendiaca@udlap.mx (R.E.B.-C.).

[3] Tecnologico de Monterrey, Escuela de Ingeniería y Ciencias, Av. Gral. Ramón Corona No 2514, Colonia Nuevo México, Zapopan 45121, Jalisco, Mexico; gildardo.sanchez@tec.mx (G.S.-A.); diegonl@tec.mx (D.E.N.-L.); alsl@tec.mx (A.L.S.-L.).

* Correspondence: jose.lozada@udlap.mx (J.D.L.-R.); eugenio.sanchez@udlap.mx (E.S.-A.); edgarl@tec.mx (E.R.L.-M.).

† These authors contributed equally to this work.

Figure S1. GC/MS chromatogram of hexane extract of *K. tomentosa*.

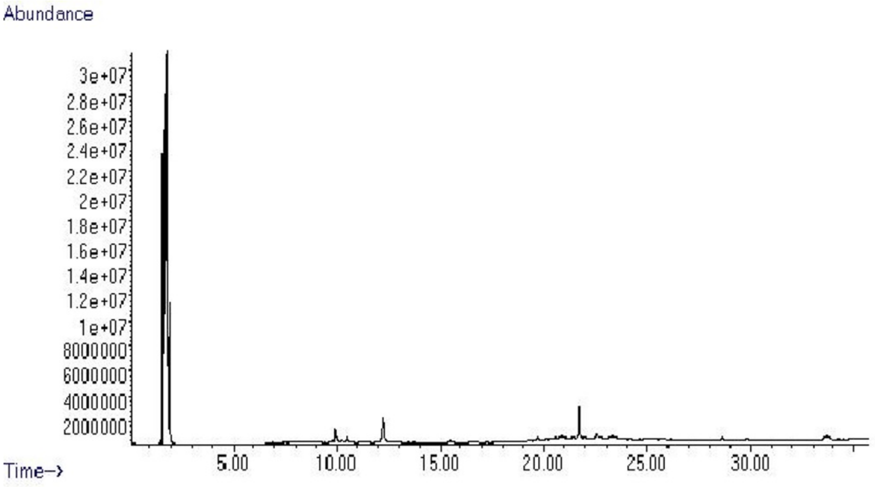

Figure S2. GC/MS chromatogram of chloroform extract of *K. tomentosa*.

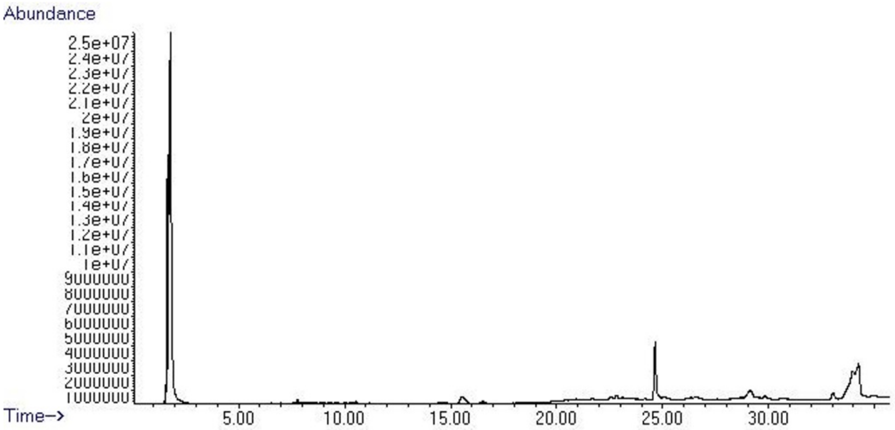

Figure S3. UHPLC/MS chromatogram of methanol extract of *K. tomentosa*.

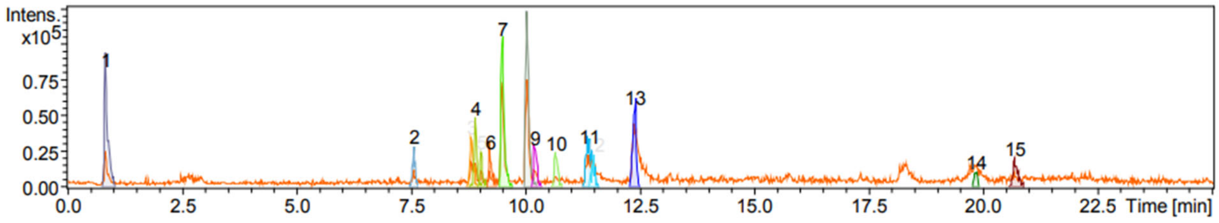

Figure S4. Docking analyses results of identified compounds in the methanol extract of *K. tomentosa*.

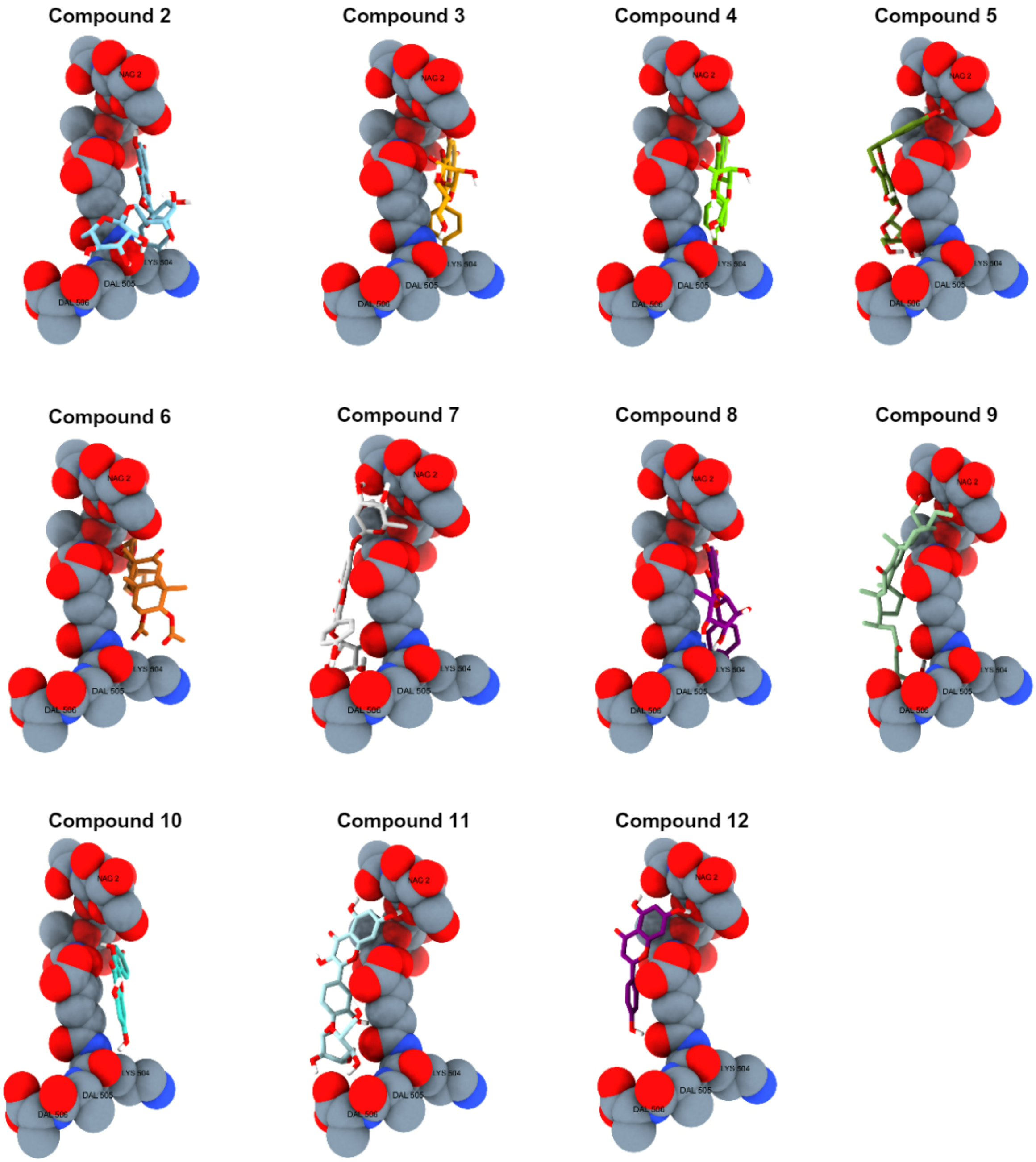

Table S1. Identified compounds in the methanol extract from *K. tomentosa* and their retention time.

Rt (min)	Compound	[M−H]⁻	Reference
0.8	N.I.		
7.6	Kaempferol-3-*O*-rutinoside	593	[43]
8.8	Quercetin-*O*-hexoside	463.093	[44]
8.9	Kaempferide-3-glucuronide	597.2	[45]
9.0	Eriodictyol-7-*O*-hexoside	449	[45]
9.2	Deacetoxy (7)-7-oxokhivorinic acid	535.2	[46]
9.5	Kaempferol-3-*O*-hexosyl-*O*-pentoside	579.13	[47]
10.0	Kaempferol 3-*O*-*α*-L-arabinopyranosyl-(1→2) *α*-L-rhamnopyranoside	563.14	[47]
10.2	Kaempferin	431.1009	[48]
10.7	Ganolucidic acid C	517	[49]
11.4	Kaempferol	285.04	[48]
11.5	Spiraeoside	301.01	[48]
12.4	Apigenin	269.045	[48]
19.8	Linoleic acid	279.2	[46]
20.7	Heliannuol A	250.14	[50]

Table S2. Binding energies of identified compounds in the methanol extract of *K. tomentosa* with the NAM/NAG-peptide subunits of *S. aureus* cell wall.

Ligand	Binding energy (Kcal/mol)	SD	*n*
Compound **2**	−6.1	0.4	9
Compound **3**	−5	0.4	24
Compound **4**	−4.8	0.3	46
Compound **5**	−5.6	0.4	20
Compound **6**	−5.1	0.1	61
Compound **7**	−5.9	0.3	20
Compound **8**	−5.5	0.3	66
Compound **9**	−6.3	0.5	35
Compound **10**	−5.1	0.1	82
Compound **11**	−5.7	0.4	29
Compound **12**	−5	0.1	93

Abbreviations: SD, standard deviation; N, cluster size.
